# Editorial: Intermolecular interaction studies in binary and higher order liquid mixtures, ionic liquids and deep eutectic solvents based systems

**DOI:** 10.3389/fchem.2025.1577620

**Published:** 2025-03-18

**Authors:** Ranjan Dey

**Affiliations:** Thermophysical Properties Lab, Department of Chemistry, BITS Pilani K K Birla Goa Campus, Zuarinagar, Goa, India

**Keywords:** intermolecular interaction, binary, ionic liquids, deep eutectic solvents, multicomponent

The thermophysical properties (Bensworth et al., 2021; El Achkar et al., 2021) of a binary and higher order liquid mixture especially those comprising Ionic Liquids (ILs) and Deep Eutectic Solvents (DES) are crucial for understanding their behavior in various industrial and scientific applications. Understanding the thermophysical properties and the nature of these intermolecular interactions is essential for optimizing the use of this binary and multicomponent mixture and consequently the usage of sustainable and greener alternative in various applications in the form of ILs and DESs, including drug delivery systems, solvent recovery processes, etc. while ensuring improved efficiency, safety, and performance in practical implementations. As of today, there are more than approximately 500 commercially available Room temperature Ionic Liquids (RTILs) with multifarious commercial and industrial applications and are used in diverse processes *viz.*, solvent extraction (Prabha Padinhattath et al., 2025), separations, heat-mass transfer, process design, etc. Thorough knowledge of the thermophysical properties of the ionic liquids and their phase behaviour with solids, liquids and gases opens up newer pathways for designing “tailor-made” ionic liquids which can be fine-tuned to carry out very specific technical applications as a designer solvent. The high thermal stability, very low vapour pressure, adjustable viscosity and water miscibility make them ideally suited environmentally benign alternatives as compared to conventional organic solvents and thereby can be termed as Green Solvents.

Deep Eutectic solvents represent the emerging “New Age Green Solvents”, owing to their very low melting points depression in the eutectic region. They are slowly gaining ground as the alternatives to the Ionic Liquids owing to their specific characteristics of being safer (Prabhune and Dey, 2023), sustainable, less hazardous (Maria Perna et al., 2020) and ‘‘greener” as compared to ILs.

The physicochemical properties of Deep Eutectic Solvents are found to be very suitable owing to their (Omar and Sadehgi, 2022) low flammability, less toxicity and thermal stability and sustainability Their applications in various industrial applications has spurred a burgeoning interest stemming from their usage in diverse industrial processes like desulfurization, pharmaceutical synthesis, (Nazar et al., 2024) anomaterial manufacturing, biocatalyst development, and gas capture to name a few. They prove to be very attractive to researchers and industry from their very specific biodegradability, ease of being prepared in commercial proportions, low cost of preparation and several other interesting features. Within a very short span of time, DESs have been employed for a large and diverse range of applications including separation processes, extraction of solvents, pharmaceutical processing, environmental protection, material synthesis etc. They are also coming out strongly as a very potential contender as an effective and efficient component for separation processes in the oil and gas industry.

The four manuscripts included in this compilation encompasses a broad spectrum of industrial applications including API-ILs in their applications for riot control agent, novel task specific Ionic Liquids for metal extraction, novel deep eutectic solvent-based surfactant (DES surfactant) for the selective and fast oxidation of alcohols to aldehydes and introduction of two new indices for molecular descriptors advances in QSPR research.

The first Chapter entitled “*Synthesis, characterization and irritant effects of nonivamide irritant riot control agent based on ionic liquids*”, authors Hongying Wang et al. investigate how Active Pharmaceutical Ingredients-Ionic liquids (API-ILs) can enhance the solubility of the drug and the bioavailability of solid drugs without a change in the structure of drug molecules. They have taken Choline Chloride (ChCl) and Citric Acid as the ions for the Ionic Liquid and synthesized pelargonic acid vanillylamide (PAVA) based ILs. The findings show that these PAVA based ILs have promising potential as novel riot control agent.

The second chapter of the Research Topic entitled “*Di-[trioctyl-(8-phenyloctyl)-phosphonium] pamoate: Synthesis and Characterization of a novel, highly hydrophobic Ionic Liquid for the Extraction of Scandium, Thorium and Uranium*” by Gradwohl et al. introduces a new, task-specific ionic liquid which finds suitable application for metal extraction with considerably reduced leaching behavior compared to similar, phosphonium-based ionic liquids. The experimental findings demonstrate the enhanced suitability of [TOPP]2 [PAM] for extracting Sc, Th and U from aqueous matrices with a high degree of efficiency.

The third Chapter of the Research Topic “*Selective and Fast Oxidation of Alcohol to aldehyde Using Novel Catalytic Deep Eutectic*” by Chalaki et al. gives an insight of a novel deep eutectic solvent-based surfactant in terms of its catalytic applications. The protocol developed in the investigation provides a facile strategy and excellent efficiency to selectively oxidize various alcohol derivatives to their respective aldehydes and ketones by making use of hydrogen peroxide in the presence of catalytic DES surfactant.

The last chapter of the Research Topic entitled “*On QSAR Modeling with Novel Degree-Based Indices and Thermodynamics Properties of Eye Infection Therapeutics*” by Rasheed et al. introduces two new indices, namely, the “first revised Randic index” and the “second revised Randic index”, for the analysis of eye infection drugs. These results demonstrate a strong association between the indices and the properties under investigation.

This Research Topic of Frontiers aims to bring forth these aspects to make it interesting for a diverse scientific community.
